# Validation of “Neurit.Space”: Three Digital Tests for the Neuropsychological Evaluation of Unilateral Spatial Neglect

**DOI:** 10.3390/jcm12083042

**Published:** 2023-04-21

**Authors:** Gemma Massetti, Federica Albini, Carlotta Casati, Carlo Toneatto, Stefano Terruzzi, Roberta Etzi, Luigi Tesio, Alberto Gallace, Giuseppe Vallar

**Affiliations:** 1 Department of Medicine and Surgery, University of Milano-Bicocca, 20126 Milan, Italy; 2 NeuroMI—Milan Center for Neuroscience, University of Milano-Bicocca, 20126 Milan, Italy; 3 MiBTec—Mind and Behavior Technological Center, University of Milano-Bicocca, 20126 Milan, Italy; 4 Specialistic Rehabilitation Unit, Neuropsychological Service, Casa di Cura Villa Barbarano, 25128 Brescia, Italy; 5 Department of Psychology, University of Milano-Bicocca, 20126 Milan, Italy; 6 Neuropsychological Service, Department of Neurology, Desio Hospital, ASST Brianza, 20900 Monza, Italy; 7 Neuropsychological Laboratory, Department of Neurorehabilitation Sciences, IRCCS Istituto Auxologico Italiano, 20122 Milan, Italy; 8 Department of Neurorehabilitation Sciences, IRCCS Istituto Auxologico Italiano, 20122 Milan, Italy; l.tesio@auxologico.it; 9 Department of Biomedical Sciences for Healthy, University of Milan, 20122 Milan, Italy

**Keywords:** digital assessment, computerized assessment, neuropsychological assessment, unilateral spatial neglect, Neurit.Space

## Abstract

Patients suffering from Unilateral Spatial Neglect (USN) fail to pay attention to, respond to, and report sensory events occurring in the contralesional side of space. The traditional neuropsychological assessment of USN is based on paper-and-pencil tests, whose data recording and scoring may be subjected to human error. The utilization of technological devices can be expected to improve the assessment of USN. Therefore, we built Neurit.Space, a modified digital version of three paper-and-pencil tests, widely used to detect signs of USN, namely: Bells Cancellation, Line Bisection and Five Elements Drawing Test. Administration and data processing is fully automatic. Twelve right brain-damaged patients (six with and six without USN) and 12 age- and education-balanced healthy participants were enrolled in the study. All participants were administered both the computerized and the paper-and-pencil versions of the tests. The results of this preliminary study showed good sensitivity, specificity, and usability of Neurit.Space, suggesting that these digital tests are a promising tool for the evaluation of USN, both in clinical and in research settings.

## 1. Introduction

Unilateral Spatial Neglect (USN) is a neuropsychological disorder, more frequent and severe after right-hemispheric lesions, resulting from cerebrovascular accidents, brain injuries or tumours [1,2,3,4]. USN is a multi-componential syndrome affecting, in most patients, the left side of the space, contralateral to the side of the right hemispheric lesion, with fewer patients exhibiting right USN after left hemispheric damage [5,6,7,8,9,10,11]. Patients suffering from USN fail to pay attention to, respond to, and report sensory events occurring in the contralesional side of space (i.e., the left side of space for right-hemispheric brain-damaged patients); such patients also fail to dynamically explore these portions of the space, by eye movements and motor effectors, as the upper limb [10]. 

The multifarious manifestations of USN [9] may be schematically summarised through a primary distinction between defective and productive manifestations. The former consists in not exhibiting the appropriate behaviour, as requested by the experimental task or by the activities of daily living, and the latter in the production of gratuitous actions or the manifestation of delusional beliefs that are inappropriate for the setting [10]. 

These manifestations are mostly detected by the clinical observation of the patients and by the patients’ performance on several neuropsychological paper-and-pencil tests [10,12,13]. Especially in the post-stroke acute stage, the head, the eyes, and the trunk of brain-damaged patients with USN are often turned towards the ipsilesional side of space. Furthermore, they may not collect the food presented on the contralesional side of a plate, and if some objects are positioned in front of them, they may not collect the ones located on the contralesional side of the space. With respect to paper-and-pencil tests, the most frequently used for diagnostic purposes are Target Cancellation, Line Bisection and Drawing tasks. Target Cancellation tasks require patients to search and mark target items presented on a paper sheet area. Only targets may be individually displayed, for instance, in the line crossing test [14], or shown together with several distractors, for instance, in the letter and bell cancellation tests [15,16]. Other tasks require patients to discriminate between full vs. incomplete targets (with a left-sided or right-sided missing portion), allowing them to distinguish between egocentric and allocentric, stimulus-based, USN [17,18,19,20]. The quantitative scoring of these tasks includes the total number of target omissions, the difference between the number of omissions on the left vs. right sides of the sheet, and the time needed to complete the test. Information about other aspects of the patients’ performance includes the type of error (namely, “defective” omissions of targets vs. “productive” manifestations) [21,22], the location of the first crossed target, the directional pattern of the patients’ exploration and the “centre of cancellation” (i.e., the scaled mean position in the horizontal, left–right, dimension of cancelled targets) [23,24]. Line Bisection tasks require patients to mark the location corresponding to the objective mid-point of several horizontal lines, different in length, typically presented with their mid-point aligned with the mid-sagittal plane of the patient’s body [25]. Typically, USN patients set the mark shifted toward the ipsilesional en7d of the line (i.e., rightward from the objective mid-point of the line in the case of patients with left USN). The error is typically larger for longer than for shorter lines [25,26,27]. In Drawing tasks patients are required to copy one or more figures presented in front of them [28], or to draw them (e.g., the hours in a clock face) from memory to a verbal command [29,30]. The patients’ performance is evaluated considering the extent of omissions (complete or partial) of details in both the left and right sides of the figure or of each object when the stimulus includes multiple objects (as, for instance, in [28]). Typically, copies and drawings by patients with left USN are inaccurate and incomplete on their left-hand side. When copying multiple-object figures, patients may omit either left-hand side objects, with reference to the mid-sagittal plane of their body (egocentric USN), or the left-hand side of each object, with reference to the object’s axis (allocentric, object-based, USN) [31].

With increasing time from the onset of a stroke or a traumatic brain injury, many USN patients recover, wholly or in part, performing better or at the ceiling (optimal performance) at paper-and-pencil testing but still exhibiting difficulty in performing activities of daily living which require adequate control of spatial attention and awareness [32]. One possible explanation is that the sensitivity of neuropsychological tests decreases over the course of recovery because patients have learned to compensate successfully for their deficits during conventional testing or because the abilities required to perform well in neuropsychological tests might not correspond sufficiently to tasks and activities in the patients’ everyday environment [32,33,34]. Growing evidence indicates that, whereas traditional paper-and-pencil neuropsychological tests may be appropriate to assess USN in the acute stages of stroke, with increasing time from the onset of the injury, computer- and touch-screen-based tasks may allow detecting USN symptoms even in patients who normally perform at paper-and-pencil tests but still report mild difficulties in everyday life activities [35]. Computerized tests can record much more information than paper-and-pencil tests (i.e., accuracy, time of execution and reaction time measures simultaneously). Stimuli may be presented in varying locations and times across trials, sessions, and sensory modalities [34,36] so that various difficulty levels can be easily implemented and possibly combined with concurrent tasks. Other measures may be recorded, such as the touches of the stimuli (i.e., the latency of each touch of the screen from the previous one; the position of each touch in X and Y coordinates on the screen; type of touched item: target or distracter [37]; record of eye movements [38]). These features, along with the addition of reaction times measures (i.e., latencies), reduce the chances for ceiling effects, particularly in stroke patients in the chronic phase (one year after stroke onset [39]). For these reasons, computer- and touch-screen-based technologies, as well as the more recent virtual and augmented reality technologies [35,40,41,42], could be a promising tool for neuropsychological assessment. 

Following the hypothesis that computerized tests can be considered an evolution of paper-and-pencil tests, the aim of the present study was to develop sensitive computerized tests, “Neurit.Space”, by building up and testing a slightly modified version of three widely used paper-and-pencil tasks (one cancellation task, one Line Bisection task and one drawing task) in a digital environment based on a touch screen and digital pens, simulating the act of filling in a paper-and-pencil set-up. Novel parameters that may better detect manifestations of USN (particularly in patients in a chronic stage, in whom a partial or full recovery of the deficit has taken place), as compared to the traditional time-honoured paper-and-pencil tests, were recorded. Furthermore, the automatic scoring procedure allows to reduce both the time spent by the clinician in this activity and the risk of human error in the scoring procedure [43,44]. Finally, the possibility of digitally archiving the participants’ performances may facilitate the sharing of information for clinical and research purposes. To validate Neurit.Space, both the digital and the paper-and-pencil versions of the tests were then administered to healthy participants and right-brain-damaged patients with and without USN, most of whom were in a sub-acute stage post-stoke.

## 2. Materials and Methods 

### 2.1. Participants

A continuous series of 12 right-brain-damaged (RBD) patients (eight females), all suffering from a cerebrovascular attack (CVA), and 12 healthy participants (seven females) voluntarily entered this study. All participants were right-handed according to the Edinburgh Handedness Inventory [45] and had a normal or corrected-to-normal vision.

**Figure 1:**
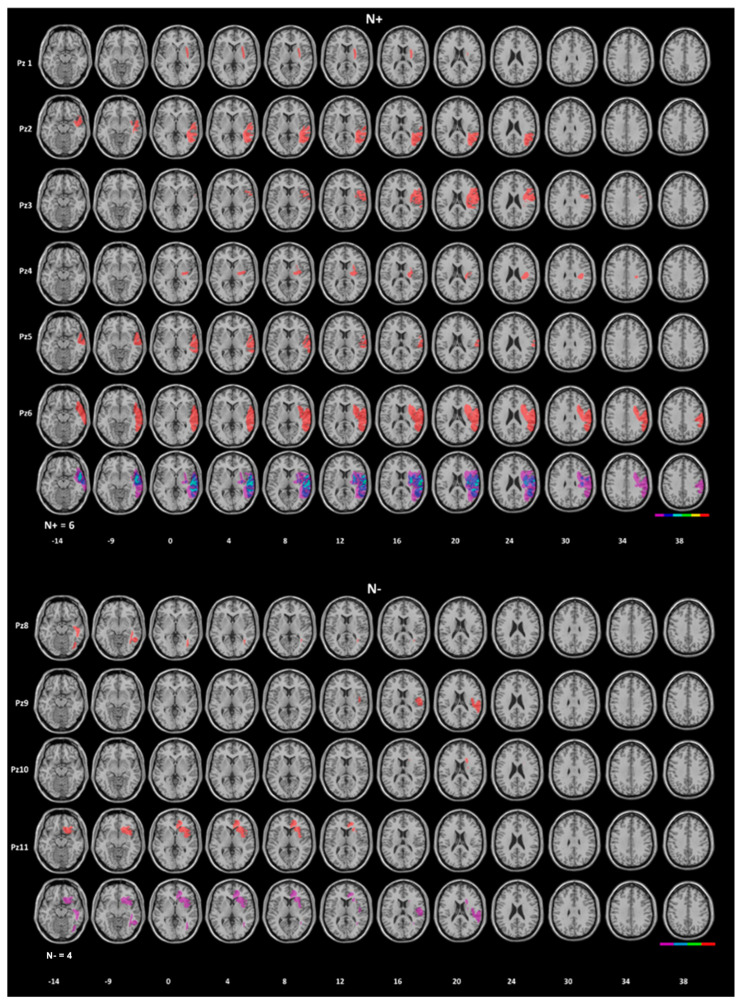
Lesion localization of brain-damaged patients. Top, N+ patients. Bottom, N− patients. Overlay lesion plots are shown (bottom row = frequencies of overlapping lesions, from dark violet, n = 1, to red, n = maximum 6). Lesions were drawn on standard MRI template with a 1-mm slice distance (voxels of 1 mm^3^). The Montreal Neurological Institute (MNI) Z-coordinates of each transverse section are reported. Right brain side on the right of the images.

Patients were recruited at the Neurorehabilitation Department of the IRCCS Istituto Auxologico Italiano, Milan, Italy. None of them had evidence or history of psychiatric disorders and showed no cognitive deterioration, assessed through the Mini-Mental State Examination, MMSE (corrected score cut-off = 22; [46]). All patients had suffered from a right-hemispheric stroke (six haemorrhagic and six ischemic). Most (eight out 12) patients were assessed in the sub-acute (one week to three months) stage of stroke (patients #1–#3, #6, #7, #9–#12), three patients (#4, #5, #9) in the successive time period, including the post-acute one (six months to one-year post-stroke); one patient (#8) was assessed in the chronic stage (one year after stroke onset) (Esposito et al., 2021 [39]). The presence/absence of USN was defined by a psychometric neuropsychological assessment. According to the patients’ performance in the Apples Cancellation Test, (Mancuso et al., 2015 [18]) (namely, at least two out of three pathological scores among total, egocentric and allocentric error scores), patients were subdivided into two groups: (i) patients with USN (RBD/N+, N = 6) and (ii) patients showing no evidence of USN (RBD/N−, N = 6). The Apples Cancellation Test was used primarily due to its higher sensitivity in detecting USN, compared to other cancellation tests (Basagni et al., 2017 [47]), and, furthermore, to its ability to distinguish both the egocentric and the allocentric components of USN (see, for instance, Ota et al. 2001 [19], 2003 [20], Bickerton et al., 2011 [17]). The patients’ demographic and clinical features are summarized in Table 1. The site of the lesions was assessed by CT or MRI Scan (Scan images were not available for two N− patients) (Figure 1). 

Healthy participants (mean age: 71.6 ± 7.39 years, range: 57–83; mean educational level: 11.17 ± 4.59 years, range: 5–18) were enrolled from experimenters’ relatives and acquaintances. They had no history or evidence of neurologic and psychiatric disorders nor reported any other acute medical condition. The two clinical groups did not differ in schooling (USN+ = 13.33 ± 4.76 years; USN− = 13.83 ± 3.87. t(10) = −0.199, *p* = 0.845). A small difference in age was found between the two groups (USN+ = 75.17 ± 11.58; USN− = 58.83 ± 13.01. t(10) = 2.296, *p* = 0.044), in line with previous studies [48].

### 2.2. Study Design

Participants underwent two different experimental sessions lasting about 20 min each, with an inter-session interval of at least 24 h. Sessions’ order was counterbalanced across the groups. In one session, participants completed three paper-and-pencil tests: (i) Bell’s Cancellation [16], (ii) Line Bisection [27] and iii) Five Elements Complex Drawing Test [28] in a randomized fixed order. In another session, participants completed the computerized tests in a randomized fixed order.

Neurit.Space was developed to run on a Microsoft Surface Laptop 3, but it can be easily adapted to other touch-screen monitors. Microsoft© Visual Studio 2019 (Net Framework using VisualBasic language) and Adobe Illustator© CC 2019 were used for graphics’ creation. To make the digital tasks as similar as possible to the classical paper-and-pencil tests, participants sat at a table with the touch-screen monitor leaning on it, aligned with the mid-sagittal plane of their trunk, and were required to complete all the tasks using a Microsoft© Surface Pen (tests can be easily adapted to other digital pens). Participants’ digital marks were made recognizable due to the use of green ink. The system collects (i) pen position (x and y coordinates in pixels) and (ii) pressure (4096 levels); Additional data include Azimuth Orientation, X-Tilt Orientation and Y-Tilt Orientation. Screenshots showing participants’ marks were automatically produced by the application as a qualitative output.

**Figure 2:**
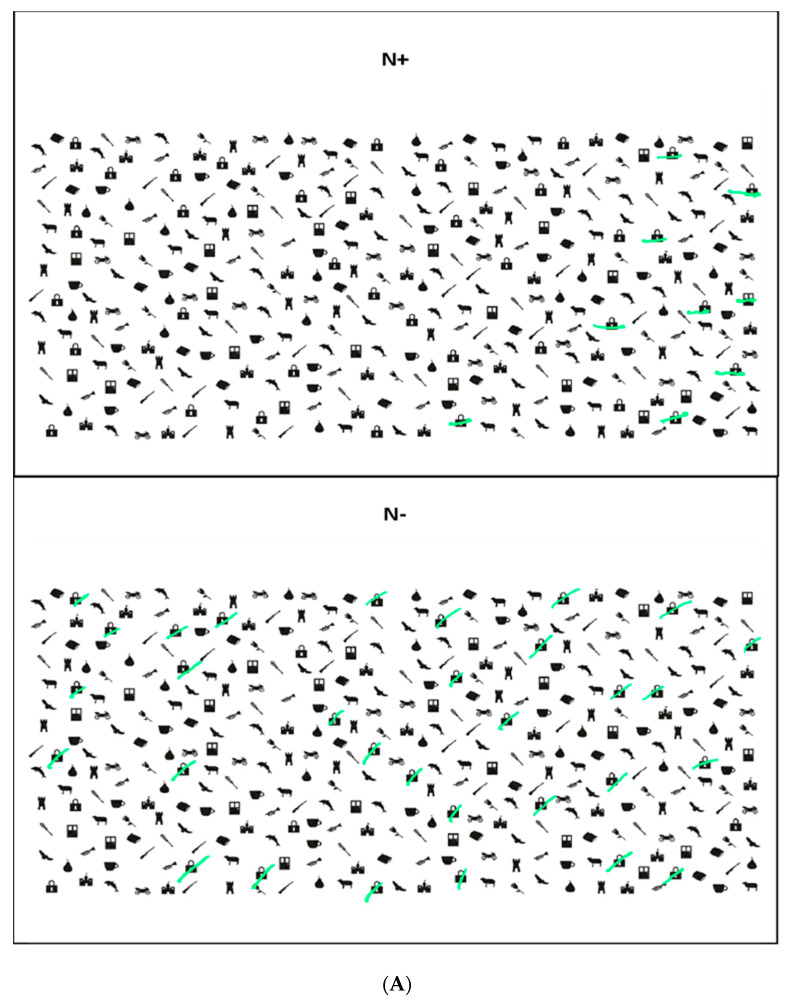

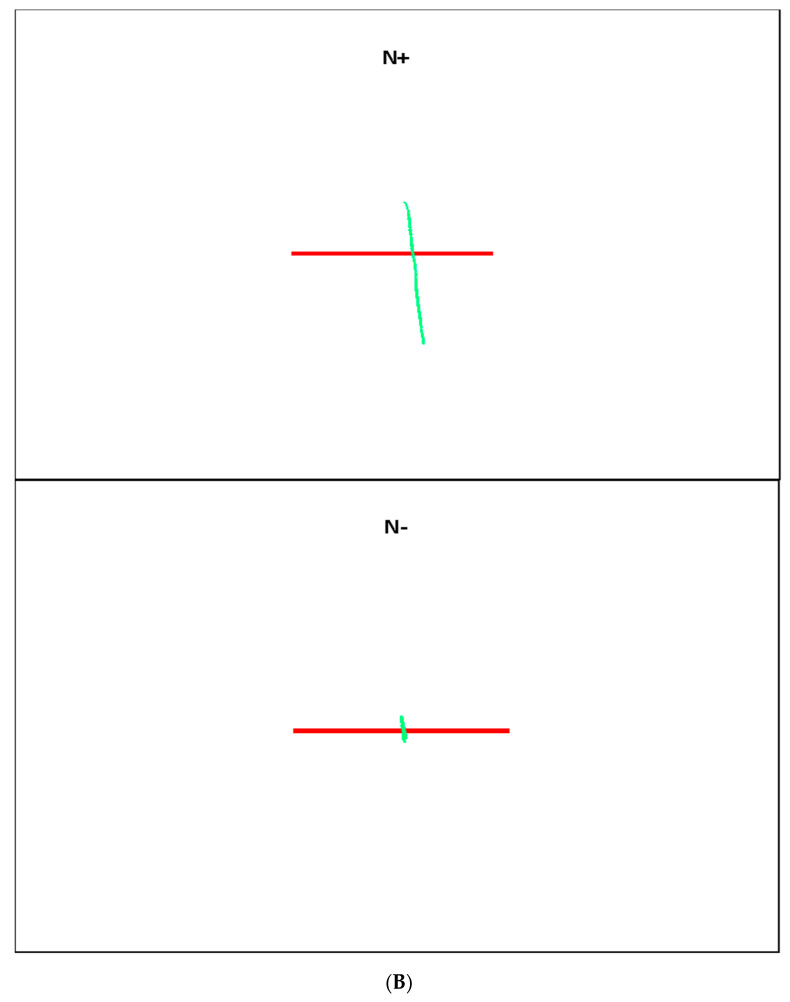

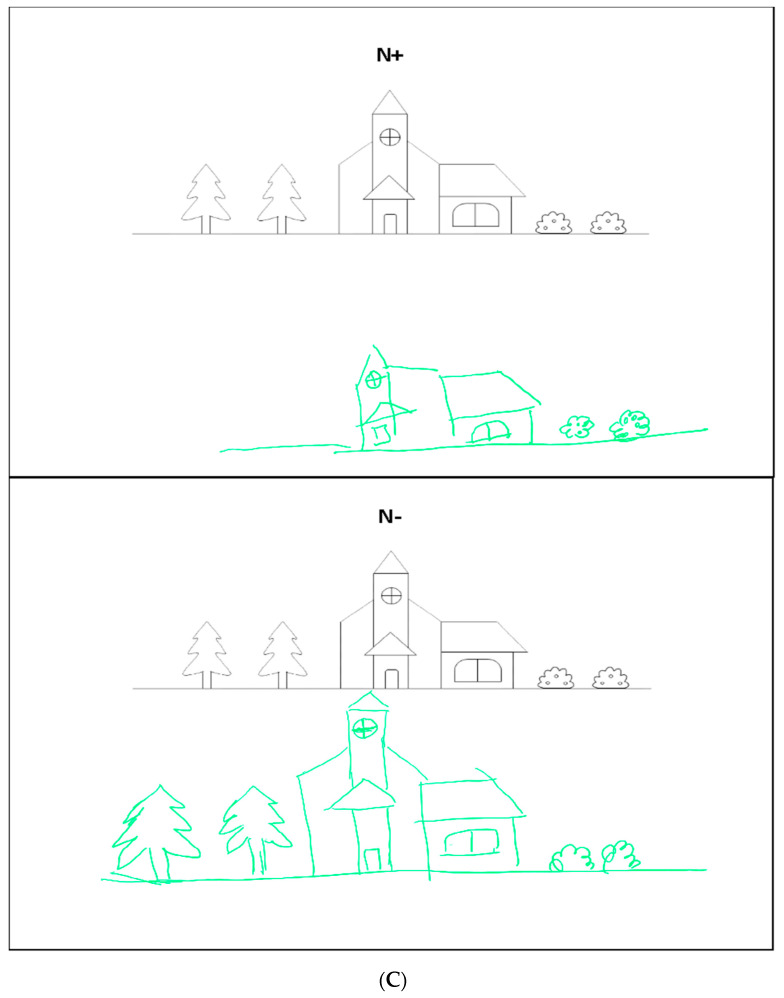
(**A**). Example of N+ (on the top) and N− (on the bottom) patients’ performance at Neurit.Space in the Padlocks Cancellation Test. (**B**). Example of N+ (on the top) and N− (on the bottom) patients’ performance at Neurit.Space in the Digital Line Bisection Task. (**C**). Example of N+ (on the top) and N− (on the bottom) patients’ performance at Neurit.Space in the Digital Five Elements Drawing Test. The green lines indicate the patients’ marks in the Cancellation (**A**), Line Bisection (**B**), and Drawing (**C**) tests.

Neurit.Space included these three digital tasks:

(i) Padlocks Cancellation Test (Figure 2A). Participants were asked to cross out all padlocks’ outlines presented on the surface of the screen among 336 distractors (i.e., outlines of doors and motorbikes). Stimuli were pseudo-randomly organized in twelve different quadrants: four on the left side, four in the middle and four on the right side of the screen. Targets and distractors were equally distributed in all of the 12 quadrants (each side of the screen contained 12 padlocks, for a total of 36 padlocks). Participants were asked to be as fast and accurate as possible and to communicate to the experimenter when they considered they had completed the task. The actual test was preceded by a practice trial with three targets and two distractors. Three different scores were assigned: (a) Total Accuracy Score (i.e., the number of targets correctly cancelled. Range: 0–36); (b) Asymmetry Score (i.e., the difference between the number of targets correctly marked in the left-hand side and the number of targets correctly marked in the right-hand side of the screen (+) and in the left one (range 0–±12); (c) Total Time to complete the test. Five other parameters were registered: (d) Time Imbalance, namely the amount of time spent on each side of the display (left vs. right); (e) Centre of Cancellation (CoC), namely: the centre of the participants’ marks in the x and y coordinates on the screen (Rorden and Karnath, 2010 [23]; Toraldo et al., 2017 [24] for a similar parameter); (f) Starting and Arrival Points, namely: the first and the last stimulus marked); (g) Errors other than omissions, such as commission errors, namely: false alarms, and perseveration, such as repeated marks and other gratuitous productions, not required by the task’s instructions (Rusconi et al. 2002 [22]). The exploration track was also available, and it could be further reviewed in a video movie action stored in the device.
(ii) Digital Line Bisection Test (Figure 2B). Participants were asked to mark the mid-point of 4 red lines (25 cm in length) displaced horizontally in the centre of the screen. The test was preceded by two practice trials. The distance between the marked subjective mid-point and the objective mid-point of the line (negative values for left shifts from the mid-point and positive values for right shifts) is automatically computed and stored.
(iii) Digital Five Elements Drawing Test (Figure 2C). Participants were asked to copy a complex figure at the top of the screen as accurately as possible, including five elements: two pine trees on the left-hand side, two bushes on the right-hand side and a church in the centre. The test was preceded by a practice trial in which participants were asked to make a free drawing (even a scribble) on a blank digital sheet to make them comfortable with the use of the electronic pen. The Total Time to complete the test and the Trajectory of drawings are recorded: participants’ performance can be further reviewed, sign-by-sign, in a video movie action. To provide a quantitative score of participants’ performances, a semi-automatic scoring procedure was used: omission errors were scored by the examiner as reported in [49]. For each element, 2 points were assigned for an errorless copy; 1.5 points for partial left/right-sided omissions, 1 point for complete left/right-sided omissions, 0.5 points for complete left/right-sided omissions and partial right/left-sided omissions and 0 points if no element was recognisable. The horizontal ground line was not considered for the scoring attribution.

### 2.3. Statistical Analyses

All the analyses were carried out with the software SPSS© (IBM SPSS Statistic version 23, IBM Corp., Armonk, NY, USA). 

To compare the scores on the two version of the tests (i.e., paper-and-pencil and digital), the raw scores for the Drawing tests (range: 0–10) were analysed. The raw scores expressed in centimetres for the Bisection tests were adopted (in the digital version, pixels are automatically converted into centimetres by an algorithm embedded in the software). For the Cancellation test, the raw scores were turned into percentage scores. Finally, the raw score expressed in seconds for the ‘Time Imbalance’ and the percentages for the ‘Centre of Cancellation’ were computed. 

Since the normality test of Kolmogorov–Smirnov violated the null hypothesis, non-parametric tests were used. The Wilcoxon Signed Rank Test was used to verify the equivalence between the paper-and-pencil and digital versions of the tests. Then, the Mann–Whitney non-parametric independent samples t-test was used to explore the presence of any difference in both the digital and the paper-and-pencil versions of the tests between the performances of N+ and N− patients, comparing the standard parameters as well as the time imbalance and the centre of cancellation. 

## 3. Results

### 3.1. Paper-and-Pencil Test vs. Neurit.Space

#### 3.1.1. Healthy Participants

**Figure 3:**
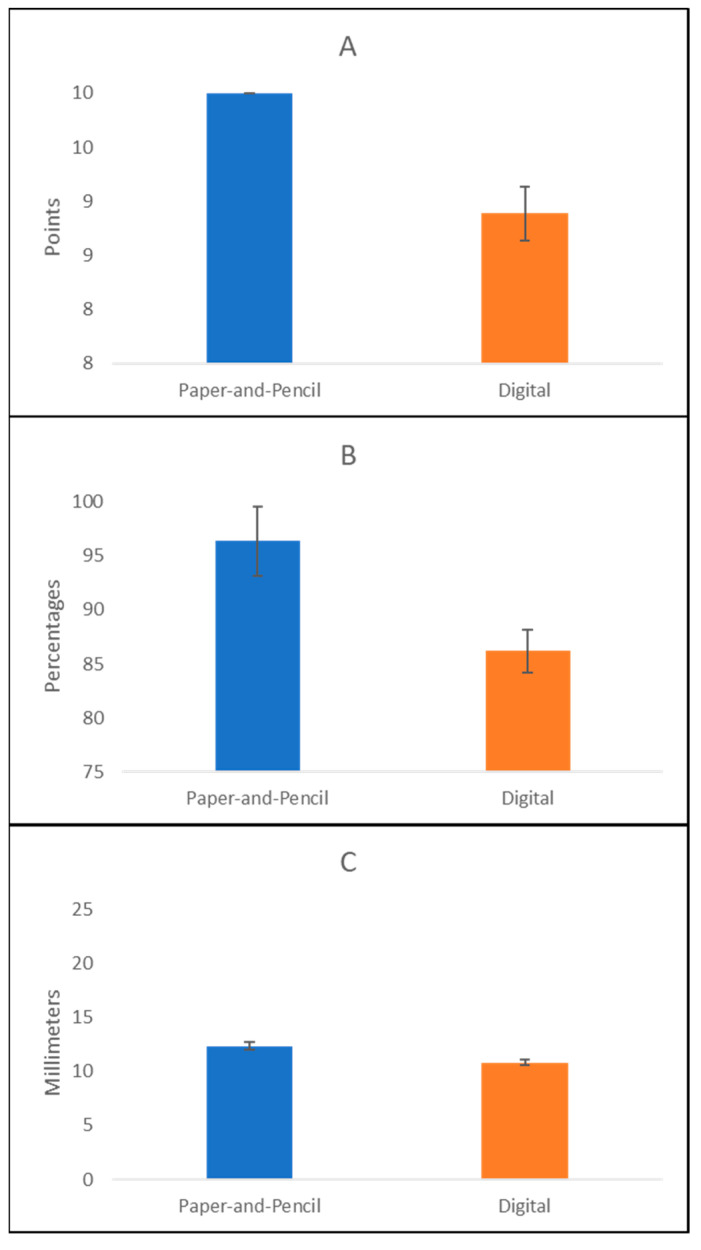
Mean performance scores in the paper-and-pencil vs. digital tests in healthy participants. Error bars represent ± 1 SD. Panel (**A**) Drawing tests; Panel (**B**) Cancellation tests; Panel (**C**) Digital Line Bisection test.

No significant difference emerged between the performance of the healthy participants on the two versions of the tests (paper-and-pencil vs. computerized; Figure 3). Particularly, the Wilcoxon Signed Rank Test showed no differences between the healthy participants’ performances on the Five Elements Drawing Test (raw score mean: 10 ± 0 points) and the Digital Five Elements Drawing Test (raw score mean: 9.87 ± 0.25 points) (z = −1.604 and *p* = 0.109). The same results were found in the cancellation tasks (total score percentage at the Bells Cancellation Test: 93.08 ± 7.35%; total score percentage at the Padlocks Cancellation Test: 96.98 ± 2.20%) (z = −1.726 and *p* = 0.084), and on the Bisection tests (mean paper-and-pencil: 11.85 ± 1.45 cm; mean computerized: 12.39 ± 0.32 cm) (z = −1.177 and *p* = 0.239). 

#### 3.1.2. Patients with USN vs. Patients without USN

**Figure 4:**
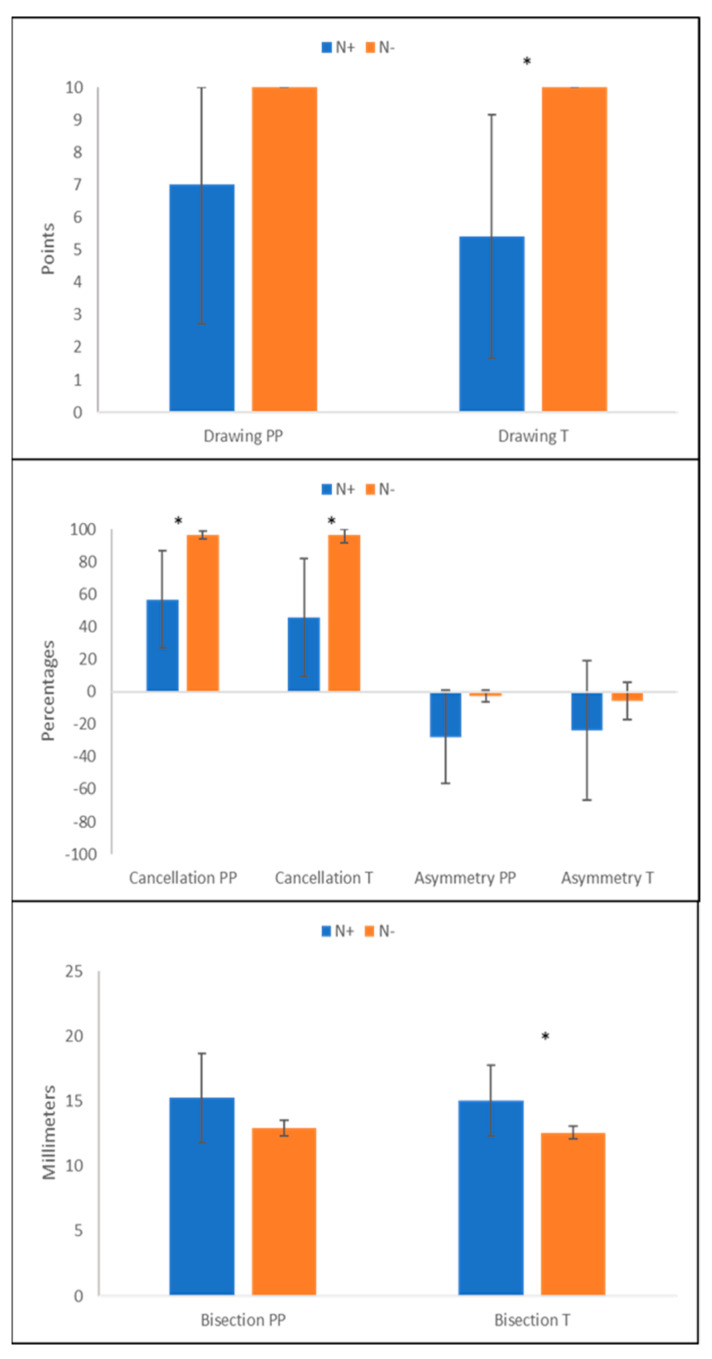
Mean performance scores for N+ and N− patients at the paper-and-pencil (PP) and computerized (T) tests. Error bars represent ± 1 SD. Asterisks represent significant differences.

Results showed that all three computerized tests discriminated between the performances of patients with vs. those without USN; on the contrary, as for the paper-and-pencil tests, only the Bells Cancellation test proved to be able to differentiate between the two groups (Figure 4). Indeed, the Mann–Whitney U test highlighted a significant difference between the performance of N+ and N− patients at the Digital Five Elements Drawing Test (N+ raw score mean: 5.42 ± 3.74 points; N− raw score mean: 9.66 ± 0.25 points) (z = −2.135 and *p* = 0.041), at the Padlocks Cancellation Test (N+ total score percentage: 56.63 ± 33.96%; N− total score percentage: 94.9 ± 5.39%) (z = −2.812 and *p* = 0.002) and at the Digital Lines Bisection Test N+ mean: 15.05 ± 2.71 cm; N− mean: 12.65 ± 0.49 cm) (z = −2.402 and *p* = 0.015). As aforementioned, these differences disappeared for the paper-and-pencil version of the Five Elements Drawing Test (N+ raw score mean: 7.00 ± 4.28 points; N− raw score mean: 10.00 ± 0.00 points) (z = −1.89, *p* = 0.180) and Lines Bisection Test (N+ mean: 15.22 ± 3.40 cm; N− mean: 12.84 ± 0.60 cm) (z = −0.727, *p* = 0.485). The only paper-and-pencil test that proved to be able to detect a significant discrepancy between patients with and patients without USN was the Bells Cancellation Test (N+ total percentage score: 56.76 ± 29.97%; N− total percentage score: 96.57 ± 2.39%) (z = −2.751, *p* = 0.004).

As for the Asymmetry score in the Target Cancellation tests, a further Mann–Whitney U test using these data was run. No significant difference was found between the performance of N+ and N− patients in both the Bells Cancellation test and the Padlocks Cancellation test (computerized asymmetry: z = −1.2, *p* = 0.25; papery asymmetry: z = −0.97, *p* = 0.33).

#### 3.1.3. Patients vs. Healthy Participants

Comparisons between patients and healthy controls showed that patients with USN scored at least two standard deviations from healthy participants, in contrast to patients without USN (Table 2). 

Patients with USN omit significantly more stimuli in both versions of the Drawing and Cancellation tests and show a rightward bias in both versions of the Bisection task.

### 3.2. Further Analyses to Deepen the Padlock Cancellation Tests: New Parameters

#### 3.2.1. Quantitative Parameters: Time Imbalance and Centre of Cancellation (CoC): Patients vs. Healthy Participants

**Figure 5:**
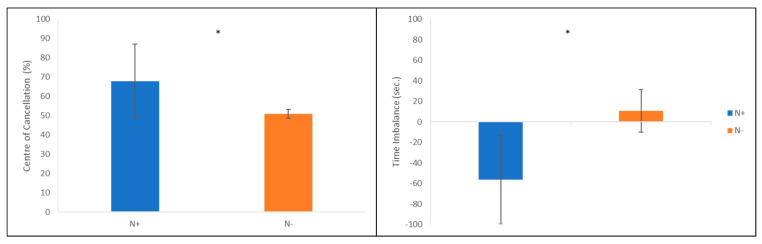
On the left, time imbalance mean scores of N+ and N− patients (seconds; positive values represent time spent on the left, while negative values represent time spent on the **right**); On the right, CoC mean scores of N+ and N− patients (percentages; values higher than 50 represent a CoC shifted toward the right, while values lower than 50 represent a CoC shifted toward the **left**). Error bars represent ± 1 DS. Asterisk represents a significant difference.

Time imbalance scores are automatically calculated. Negative values represent an imbalance toward the right side of space. Analyses showed significant differences between N+ and N− patients’ performances (Figure 5): patients with USN spent significantly less time exploring the left side of the worksheet and more time on the right side; on the contrary, patients without USN spent most of the time exploring the left side (N+ mean: −56.46 ± 43.20 s; N− mean: 9.23 ± 20.85 s) (z = −2.722 and *p* = 0.004). Furthermore, the patients’ mean differed by at least two standard deviations from that of healthy participants (8.57 ± 11.53 s; Table 3).

Centre of Cancellation scores were automatically calculated, too, through the coordinates X (horizontal axis) and Y (vertical axis) expressed in percentages, calculating the midpoint of participants’ marks on the worksheet. For the purposes of this study, only the values of the X-axis were considered: a score of 50% indicated a midpoint corresponding to the exact centre of the worksheet; negative values represented a centre shifted to the right. Analyses showed a trend in the comparison between the Centre of Cancellation marked by patients with vs. without USN (Figure 5). In fact, the CoC of USN patients was shifted toward the right, although its position was not significantly different from the one of N− patients. Mann–Whitney U test highlighted an asymptotic significance equal to p = 0.055 and an exact significance equal to p = 0.65 (z = −1.922) indeed. Finally, a descriptive comparison among N+ patients, N− patients and healthy participants showed that N+ patients had a CoC shifted toward the right (mean: 67.91 ± 19.02%), which differs by at least two standard deviations from the ones of N− patients (mean: 50.81 ± 2.25%) and healthy participants (mean: 49.51 ± 1.09%; Table 3).

##### Clinical Application of the New Quantitative Parameters: Patient #9

To explore the clinical application of the new quantitative parameters (Time imbalance and Centre of Cancellation), we further analysed the performance of patient #9, who belonged to the group of patients without USN, due to her results in the paper-and-pencil tests, which were in line with normative data (Mancuso et al., 2015 [18]). Patient #9 obtained a Time Imbalance score = −18.19 s and a Centre of Cancellation = 54.61%. These outcomes collocate her in a mid-position between patients with and without USN. In fact, we analysed her as a single case through a modified *t*-test (Crawford and Howell, 1998 [50]), obtaining the following results: in the comparison between patient #9 and the group of patients with USN, we obtained no significant difference both for the Time Imbalance (*p* = 0.44) and for the Centre of Cancellation (*p* = 0.54). Parallelly, we obtained no significant difference between patient #9 and the group of patients without USN, both for the Time Imbalance (*p* = 0.06) and for the Centre of Cancellation (*p* = 0.17).

#### 3.2.2. Qualitative Parameters: Starting Point and Other Errors: Patients vs. Healthy Participants

**Figure 6:**
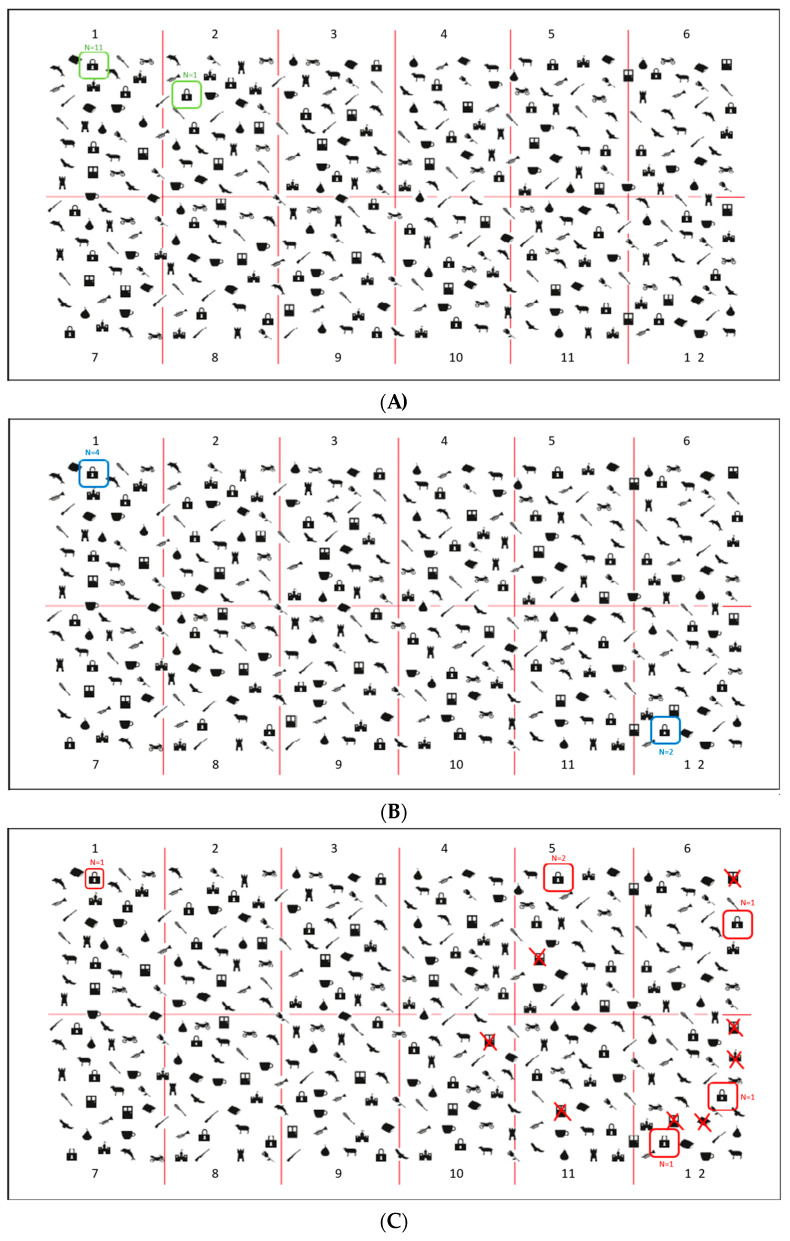
Padlock Test. Panel (**A**) (green square). Healthy participants starting point: 11 healthy participants started from the first upper left quadrant, 1 from the second upper left quadrant. Panel (**B**) (blue square). Patients without USN starting point: four patients without USN started from the first upper left quadrant, two from the twelfth lower right quadrant. Panel (**C**) (red square). Patients with USN starting point: two patients with USN started from the twelfth lower right quadrant, one from the sixth upper right quadrant, two from the fifth upper right quadrant, and only one from the first upper left quadrant. Other items marked with X = Other Errors; only patients with USN committed such errors Panel (**C**).

As for the Starting Point, five out of six patients with USN started from the right side of the worksheet, while all healthy participants and four out of six patients without USN started from the left side of the worksheet (for details, see Figure 6).

As for the Other Errors, four out of six patients with USN committed at least one such error. On the other hand, neither N− patients nor healthy participants made these errors.

### 3.3. User Experience and Usability

The System Usability Scale (SUS) [51] was used to evaluate the user experience of Neurit.Space from the healthy participants and from five expert clinicians using it. Results showed very good usability ratings from both healthy participants (mean: 86/100) and clinicians (mean: 70/100). 

Furthermore, none of the patients reported any discomfort or adverse reaction related to using the touch-screen monitor and an electronic pen instead of a classical paper sheet with a normal pen. As for the user experience, opinions collected through the think-aloud method [52,53,54] reported better visibility in the digital version of the tests thanks to a higher quality of the graphic details, especially in Padlocks Cancellation Test, and the screen’s retro-illumination. Lastly, healthy participants also claimed that it was easier to find the midpoint during the digital version of the Line Bisection Test than in the paper-and-pencil version.

## 4. Discussion

The recent literature points out that, with increasing time from the onset of a brain injury, such as a stroke, most USN patients may perform within the range of normal/unimpaired performance at paper-and-pencil testing but still exhibit difficulties in activities of daily living [32]. Whereas classical paper-and-pencil neuropsychological tests may be acceptable to assess USN in the acute stages, with increasing time from the onset of the injury, computer- and touch-screen-based tasks may allow detecting USN symptoms more efficiently, even in patients who normally perform paper-and-pencil tests [32,33,34,35,55].

Therefore, the present study aimed at validating Neurit.Space, by comparing the performances of patients with or without USN with those of healthy control participants in three classical paper-and-pencil tests (Line Bisection, Bells Cancellation and Copy of a Complex Drawing) and in their modified digital versions (Digital Line Bisection, Padlocks Cancellation Test and Five Elements Drawing Test).

First, to verify if the new digital tests could be comparable to the “gold standard” paper-and-pencil tests, the performances of a group of healthy participants at both versions of the tests were compared. Results showed no significant differences in performances, in line with the recent literature suggesting that digital tests are comparable to the traditional, analogic, paper-and-pencil tests [56,57], if not more accurate [58,59,60].

Analyses also explored the presence of any differences in the performances between patients with vs. without USN in both the traditional and the digital tests to verify if Neurit.Space was more sensitive than (or equal to) paper-and-pencil testing in detecting differences in right brain-damaged patients. Results showed that Neurit.Space can highlight differences between patients with and without USN. On the other hand, among paper-and-pencil tests, only the Bells Cancellation Test [16] could discriminate between patients. In fact, extant literature suggests that cancellation tests are the most sensitive to manifestations of USN [61] and have better test-retest reliability [62] and ecological validity [63] than other typologies of tasks. Moreover, since findings from Neurit.Space, independent of the kind of task, proved to be comparable to results obtained with the paper-and-pencil tasks, it is possible to hypothesise that the present modifications (i.e., the asymmetry and the higher number of details in the copy task and the colour of the line in the bisection task) made our versions more complex and, therefore, possibly more ecologically valid, as compared to the traditional, time-honoured tests. Accordingly, our digital tests may be more sensitive than their paper-and-pencil corresponding versions. This hypothesis can be supported by the fact that, in each of the three tests, healthy participants obtained a performance mean that was at least two standard deviations above that of patients with USN, while this did not occur in the case of patients without USN.

Furthermore, the Padlock Cancellation Test recorded two new quantitative parameters: the Time Imbalance and the Centre of Cancellation. As far as the former is concerned, results showed a significant difference between the two groups of patients, highlighting that those affected by USN spent more time exploring the right side of the screen than those without USN. Moreover, patients without USN had a performance comparable to that of healthy controls, while patients with USN differed at least by two standard deviations from them. This new parameter could then be useful to detect patients with “covert neglect” [64], that is, a residual USN well compensated by patients in the paper-and-pencil assessment [32,33,34]. In fact, deepening the performances of patients without USN, we could find at least one case (patient #9) that, despite his adequate scores in all papery tests, obtained a negative time imbalance (TI = −18.19 s), highlighting that he spent more time exploring the right side of the screen. Therefore, qualitatively, it is possible to hypothesise that the patient suffered from a mild USN, “silent”, as paper-and-pencil tests are concerned. These results show then the importance of the availability of more accurate and sensitive parameters compared to those offered by paper-and-pencil tests. In fact, a cognitive deficit, although mild, could lead to a decreased degree of safety for the patient in the domestic or outdoor environment during activities of daily living [65,66,67]. For what instead concerns the Centre of Cancellation, analyses revealed a trend in the comparison between patients with vs. without USN, although not statistically significant. Again, patients without USN had a level of performance like that of healthy controls, while patients with USN differed at least by two standard deviations from them, showing a centre of cancellation shifted toward the right, in line with previous studies [23]. Newly, patient #9 displayed a centre of cancellation slightly shifted to the right side of the worksheet (X = 54.61%): a hypothetical clue of “silent” neglect.

Finally, our system also registered two qualitative parameters: the Starting Point and the Other Errors. The first was obtained from the exploration track, while the second was from the distractors erroneously marked by participants, as well as any other commission error. Patients with USN showed qualitatively different behaviours in both parameters compared to patients without USN and healthy controls. This result is in line with current literature, which has repeatedly highlighted that patients with USN tend to start exploring the space from the right side of the worksheet and to commit such productive errors on that side [9,21,22].

Another important aspect of the present study was the assessment of the user experience of Neurit.Space, along with the choice of adopting a tablet and a digital pen. Results showed a good index of usability, a better visibility guaranteed by more precise graphic details and no adverse reactions. Neurit.Space proved to be as easy to be used as traditional paper-and-pencil tests. Furthermore, our tests obtained excellent evaluations from the clinicians: contrary to their paper-and-pencil counterparts, digital tests provide the possibility to watch a replay of the performance, allow to save physical space and spare paper sheets (in line with the idea of adopting a sustainable approach also in clinical assessment), offer the possibility to access the database remotely, easily sharing data among professionals, guarantee that the assessment is not influenced by environmental factors, as quality printing or natural lighting, since the constant retro-illumination and resolution of the screen decreases the probability of human error, also automatizing the scoring calculations. Lastly, Neurit.Space proved to be manageable, offering the possibility to be adopted also for the patients’ bedside evaluations, such as in the acute stage of stroke.

On the other hand, the present preliminary study did not analyse the impact of the duration of the disease (i.e., the number of days after stroke onset on the patients’ performances. No patient was tested in the acute and one in the chronic stage of stroke. Most patients were tested in the sub-acute (n = 8) and in the post-acute (n = 3) stages of stroke. Future studies should focus on the effects of duration of disease on the patients’ performances in the Neurit.Space digital battery for the assessment of spatial neglect and constructional apraxia [68] by the Copy of a Complex Drawing test. Furthermore, they should investigate the minimal detectable change (MDC), which is the minimal change that falls outside the measurement error in the score of an instrument used to measure a symptom [69].

Overall, Neurit.Space appears to be a promising tool for the assessment of USN, showing preliminary good sensitivity, specificity, and usability. Moreover, the digital Padlocks Cancellation Test provides additional data, otherwise almost impossible to collect, that can enrich the diagnostic process, increasing the precision of the evaluation; this could play a key role in detecting cases of “silent” neglect. Furthermore, the present digital tests may also be used to evaluate visuo-spatial abilities in other neuropsychological disorders, such as constructional apraxia [68] and deficits of sustained and selective attention [70]. These results encourage the collection of normative data to adopt the present tests in clinical practice.

## Figures and Tables

**Table 1:** Patients’ demographic and clinical features. Lesion aetiology (I: ischemic; H: haemorrhagic). Lesion site (F: Frontal; P: Parietal; T: Temporal; O: Occipital; Ic: Internal Capsule; In: Insula; Cn: Caudate Nucleus; Th: Thalamus. R: right. +/− = presence/absence of deficit.

N	Sex/Age (Years)/Educational Level (Years)	Duration of the Disease (Days)	Lesion Site/Aetiology	Apples Cancellation Test			USN
				Total Score	Egocentric Score	Allocentric Score	
1	F/53/13	48	R Cn/CVA-H	+	+	−	+
2	F/83/5	19	R P-T-In/CVA-I	+	+	+	+
3	F/80/13	12	R F-In/CVA-I	+	+	−	+
4	F/72/18	171	R Th/CVA-H	+	+	−	+
5	M/80/13	166	R F-In-T/CVA-I	+	−	+	+
6	F/83/18	67	R T-P/CVA-I	+	+	−	+
7	F/65/8	31	Ic-Cn/CVA-I	+	−	−	−
8	M/48/13	2235	R T-O/CVA-H	−	−	−	−
9	F/59/16	133	R F-T-P/CVA-H	−	−	−	−
10	M/39/11	45	R Ic/CVA-H	−	−	−	−
11	F/70/17	23	R F/CVA-H	−	−	−	−
12	M/72/18	39	R M/CVA-I	−	−	−	−

**Table 2:** Means (M) and standard deviations (SD) of the performances of healthy participants and patients with USN (N+) and without USN (N−) in the papery and digital tests. M of N+ patients ± 2 SD, compared with the healthy participants’ M, are highlighted in red.

	Healthy Participants	N+	N−
**Paper-and-Pencil**			
Drawing Copy (points)	10 ± 0.00	7 ± 4.28	10 ± 0.00
Cancellation (%)	93.08 ± 7.35	56.76 ± 29.97	96.57 ± 2.39
Bisection (cm)	11.85 ± 1.45	15.22 ± 3.40	12.84 ± 0.60
**Neurit.Space**			
Drawing Copy (points)	9.87 ± 0.25	5.41 ± 3.74	9.66 ± 0.25
Cancellation (%)	96.99 ± 2.2	56.63 ± 33.96	94.9 ± 5.39
Bisection (cm)	12.39 ± 0.32	15.05 ± 2.71	12.65 ± 0.49

**Table 3:** Means ± standard deviations of the performances of healthy participants and patients at the new parameters of the computerized tests (time of imbalance and CoC).

	Healthy Participants	N+	N−
**Neurit.Space**			
Time Imbalance (s)	8.57 ± 11.53	−56.46 ± 43.2	9.23 ± 20.85
Centre of Cancellation (%)	49.51 ± 1.09	67.91 ± 19.02	50.81 ± 2.25

## Data Availability

Datasets associated with the current study are available from the corresponding author upon request.
